# Accountability and interactional inequality: the management of problems of interaction as a matter of cultural ideals and ideologies

**DOI:** 10.3389/fsoc.2023.1204086

**Published:** 2023-06-19

**Authors:** Melisa Stevanovic

**Affiliations:** Faculty of Social Sciences, Tampere University, Tampere, Finland

**Keywords:** conversation analysis, accountability, interactional violations, problems of interaction, interactional inequality

## Abstract

In the existing sociological literature, the notion of accountability is seen both as a tool of sense-making (intelligibility side of accountability) and as a way of maintaining larger social order (normativity side of accountability). This paper points to drastically different ways of treating an interactional violation, depending on the precise framework within which the accountabilities associated with the violation are interpreted. The normative side of accountability involves the idea of interactional inequality—that is, the notion that people are not equally held accountable for their interactional violations. I suggest that such inequalities are strengthened by the prevailing cultural ideals and ideologies of interaction according to which a competent participant can solve interactional problems as they emerge. Problems of interaction are therefore commonly let pass, and if addressed, likely to be interpreted within the framework of intelligibility. This means that the violators are likely to get away from being held accountable in the normative sense of the term. As a result, I argue, many interactional problems are commonly beyond effective intervention. In its focus on the intelligibility side of accountability CA has, not only trouble addressing interactional inequalities, but it may also inherently undermine the severity of the inequalities to be addressed. A more critical, socially and societally relevant CA would thus benefit from a more explicit engagement with the normative side of the notion.

## Introduction

Conversation analysis (CA) is specialized in the analysis of how people use turns at talk and other behaviors to implement social actions (e.g., requests, proposals, and invitations) and how these actions are organized within and across interactional encounters (e.g., Heritage, 1984). This includes the consideration of how social actions are designed to be intelligible and how that intelligibility is maintained in sequences of initiating and responsive actions.

Whereas a focus on the intelligibility of action fits well with the ethnomethodological policy of indifference, it is not enough for a social scientist seeking to exercise social and societal critique. Thus, to make a positive change in the world, a CA researcher faces the need to incorporate normative notions into their inquiry. In this paper, I explore the notion of *accountability* in this regard, pointing to drastically different ways of treating an interactional violation, depending on the precise framework within which the accountabilities associated with the violation are interpreted.

In the two main sections below, I will first discuss the notion of accountability in interaction, which is followed by the consideration of people's ways of managing problems of interaction. In both sections, I will argue for the existence of what may be termed *interactional inequality*, which is suggested to be reinforced by the existing cultural ideals and ideologies regarding the management of problems of interaction. Finally, I will reflect on the position of CA in this broad field of cultural meaning-making, also considering the possibilities of a CA researcher to address interactional inequality.

## Accountability in interaction

In the existing sociological literature, accountability has been seen both as a tool of sense-making (*intelligibility* side of accountability) and as a way of maintaining social order (*normativity* side of accountability).

The intelligibility side of accountability has constituted a central focus of CA. According to Garfinkel (1967), “the activities whereby members produce and manage settings of organized everyday affairs are identical with members' procedures for making those settings ‘accountable”' (p. 1). Accountability is thus seen both as a starting premise and a core principle of inquiry (Koschmann, 2019). It means that social actions are already by virtue of their mere occurrence rendered intelligible—that is, mutually observable, describable, and explicable. In CA, such intelligibility is considered to emerge through a “sequential architecture of intersubjectivity” (Heritage, 1984), which relies on participants being able to orchestrate specific interactional practices to make their actions intelligible (or “account-able”)—that is, recognizable and understandable as, say, requests, proposals, offers, or complaints (Schegloff, 1995; Levinson, 2013).

However, the status of an utterance or other behavior as a specific action is never more than one “possible” action among multiple possibilities. Whenever participants are challenged for their actions by their co-participants, they may deny having intended their conduct to be interpreted in that specific way. This holds even to most conventional practices to implement specific actions (Robinson, 2016, p. 9–11). According to Garfinkel (1967), a specific action, such as “an agreement, as of any particular moment, can be retrospectively reread to find out in light of present practical circumstances what the agreement ‘really' consisted of” (p. 74). In other words, participants can always *post hoc* manipulate the status of their prior actions in line with their current goals and aims.

The production of intelligible courses of interaction has a normative dimension to it. Prior actions impose variably rigid normative constraints for actions to come, while a failure to produce what is normatively expected is a violation to be treated as accountable (Heritage, 1984, p. 245–253)—that is, the violator may need to provide an account for their failure. The accounts serve the maintenance of the normative organization of interaction in that they present the violations as “exceptions that prove the rule” (Heritage, 1988, p. 140). Essentially, however, it is the expectations of future accountability that guide participants' behavioral choices in the present. As pointed out by Hollander (2018), “only when people's behavior deviates significantly from what is expected are they actually called to account for it; most of the time, they discipline themselves through the anticipation of potential consequences” (p. 177).

Importantly, the normative side of accountability is not only about risking a misunderstanding or casting doubt on a person's status as a competent communicator. In addition, it encompasses people's claims of rights and obligations (e.g., Stevanovic and Peräkylä, 2014), which, in turn, are linked to social identity categories (West and Zimmerman, 1987, p. 135–136). Thus, for example, when a boy is teased by being called a “sissy”, this labeling triggers an “accountability ritual” (Cook, 2006), in which the boy must provide evidence that he indeed belongs in the social category of a “male”—or be excluded from social acceptability (Hollander, 2018, p. 178). The interactional endorsement of the identity claims is thus highly consequential for the participants in a long run (Schwalbe, 2008; Hollander, 2018).

The normative side of accountability is thus intertwined with power (Scott and Lyman, 1968; Cook, 2006). The powerful are shielded from accountability demands, while they can hold others accountable for their actions (West and Fenstermaker, 2002, p. 541; Hollander, 2018, p. 178). From this perspective, accountability is also a locus of, and a mechanism that serves to maintain, inequality—as famously clarified by Schwalbe (2008) in his introduction of the notion of “nets of accountability”. Sometimes people may be caught in several conflicting accountability structures, such “labyrinths of accountability” supporting dominant ideologies of social hierarchy (Cottingham et al., 2016).

## Managing problems of interaction

Accountability in interaction becomes apparent when problems occur. The CA notion of “repair organization” refers to the routine ways in which participants deal with problems of speaking, hearing, and understanding (Schegloff et al., 1977; Robinson, 2006; Dingemanse et al., 2015). While most trouble is resolved within the same turn of talk by the same participant whose talk embodies the trouble (Schegloff et al., 1977), co-participants may also initiate repair through various practices, such as open requests (e.g., *Huh?*), more restricted repair initiations (e.g., *Who?*), and offers of candidate understanding (e.g., *She had a boy?*).

People may, however, also choose *not* to address the problem in any way. As pointed out by Schegloff (2000), people let the problem pass in hope that “things said subsequently will clarify the problem and avoid the need to initiate repair, and if they don't, then you can ask later on when it's next relevant” (p. 116). The let-it-pass strategy serves to maintain *progressivity*. Schegloff (2007) suggested that any element that intervenes between some element and what it projects “will be heard as qualifying the progressivity of the talk and will be examined for its import” (p. 15). Although progressivity is by far not the only concern that participants orient to, it has often been observed to take priority over other concerns, such as intersubjectivity and mutual understanding. This has been found to be the case, for example, when referring to persons or places (Heritage, 2007) or when communicating with participants with interactional deficits, such as autism (Sterponi and Fasulo, 2010) or aphasia (Perkins, 2003). Furthermore, in multi-party interaction participants have been observed to orient to questions as needing a prompt answer, even if the production of the answer would override the right of the selected next speaker to provide it (Stivers and Robinson, 2006).

If participants tend to use the let-it-pass strategy to manage problems of speaking, hearing, and understanding, this is even more so when the problems have to do with the implicit claims of—and the co-participant's lack of endorsing—rights and obligations associated with the participant's identity. Such problems of interaction have been found to often take the form of such subtle violations of expectations that they practically circumvent any explicit accountability demands (e.g., Stevanovic and Peräkylä, 2014; Stevanovic, 2018, 2021). In addition to the inherently intangible nature of these types of violations, the possibilities to address them are specifically difficult for those who suffer the most from these violations. In their theory of interactional disruption, Tavory and Fine (2020) suggested the capacities of participants to disrupt interaction are distributed unequally, following the social distribution of power and authority. The unequal distribution of the power to disrupt can, for example, clarify the structures of conversational interruption (Zimmerman and West, 1975), school bullying (Evans and Eder, 1993) and sexual harassment (McLaughlin et al., 2012). What is essential is the ability of the powerful to break the ritual expectations with respect to an interactional encounter with relative impunity, while the powerless simply let it pass (Tavory and Fine, 2020, p. 380–381).

**Figure 1:**
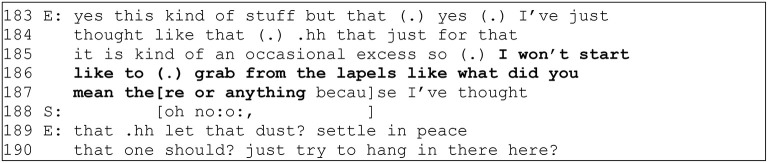
C65_36:23 (drawn from Stevanovic et al., forthcoming).

If interactional violations are difficult to address immediately in the primary encounter, there is still the theoretical possibility for the participants to account for the problematic interactional experience in retrospect. Indeed, the production of such accounts to third parties is elementary for others to be able to evaluate the problematic situation and intervene if needed. However, the production of such accounts is a complex endeavor. The interactional violations may be generally difficult to “document” in a credible way (Acker, 2006, p. 451). Single incidents may come across as too trivial to raise (Valian, 1999; Krefting, 2003) but complaining about a common occurrence may highlight the complainer's inability to accept just how things are (Gill et al., 2017, p. 1). Furthermore, the management of interactional problems is a matter of cultural ideals and ideologies, which postulate that *whenever an interactional problem arises, a competent participant can intervene immediately*. If a participant has failed to do so and now seeks to address the problem in retrospect, they orient to a need to account for their failure of not addressing the problem immediately, as exemplified in the data extract above (see Figure 1), in which a female employee has previously reported an experience of gendered dismissal to her supervisor but now undermines the organizational relevance of her problem.

It is this mechanism—I argue—that explains the difficulties of the powerless to address interactional violations. I suggest that the mechanism works in the following way—each of the three points below constituting a hypothesis that may be subjected to empirical testing:

1. While the general preference for progressivity in interaction discourages any person to address their problematic interactional experiences, it is the powerful who control the interactional agenda and have the primary rights to disrupt the anticipated structure of the encounter. This means that, in the here and now of the interaction, the powerless are likely to let the violations pass.
2. If the violation *does* get addressed in the local context of the encounter, the violation is likely to be interpreted within the framework of intelligibility—that is, the violation can be clarified with reference to a misunderstanding and/or a problem in the given participant's communicative competence (i.e., communication skills). This means that the violator is likely to get away from being held accountable in the normative sense of the term.
3. The retrospective accounts of problematic experiences get compromised if the tellers orient to a need to present themselves as having been able to address the problem in the primary interactional event but—for some reason—chosen not to do so. Interactional inequalities are thus beyond effective intervention—not only because such problems would be difficult to address—but also because the victims of the violation themselves end up undermining the need for external intervention.

## Conclusions: addressing interactional inequality

The normative notion of interactional inequality involves the idea that not all participants in interaction are similarly held accountable for their interactional violations. I suggested that such inequalities are strengthened by the prevailing cultural ideals and ideologies of interaction, which postulate that interactional problems should be addressed as soon as they occur. I argued that inequalities are maintained through a self-reinforcing cycle in which the powerless are told to account for their problematic interactional experiences *in situ*, while their failing to do so also compromises their capacities to account for these experiences in retrospect. In this way, interactional inequalities lead to ever greater inequalities, while the powerful are increasingly shielded from accountability demands.

How can interactional inequality then be addressed—if not by the participants themselves, then at least in and through research? I maintain that such efforts necessitate a better understanding of the cultural ideals and ideologies of interaction that shape people's ideas of what a competent person should (be able to) do when interacting with others—as it were, independent of their social identity positions. A focus on communication skills training as a solution to interactional problems obscures the power-related nature of those interactional violations that certain identity populations encounter daily, while a person's inability to fill the expectations of a competent person may cause an aggregate burden, which only adds to the primary disempowering experience. Addressing interactional inequality would thus first necessitate that these mechanisms be elucidated.

Given its focus on the intelligibility side of the notion of accountability, CA has difficulties addressing interactional inequalities—a weakness that has been pointed out already in the 1990's (Wetherell, 1998; Billig, 1999a,b). Even more, there is a risk that an (over)emphasis of the intelligibility side of the notion already itself contributes to the maintenance of interactional inequalities by inherently undermining the severity of the inequalities to be addressed through the prioritization of a power-neutral grasp of the problems. Thus, a more critical, socially and societally relevant CA would benefit from a more explicit engagement with the normative side of the notion of accountability. Such an engagement calls for complementing CA's primary empirical analysis of interactional phenomena with analysis of secondary data sets (e.g., retrospective accounts of interaction) and with extensive theorizing of the normative aspects of interaction.

## Author contributions

The author confirms being the sole contributor of this work and has approved it for publication.
